# CleanCap M6 inhibits decapping of exogenously delivered IVT mRNA

**DOI:** 10.1016/j.omtn.2025.102456

**Published:** 2025-01-17

**Authors:** Zachary F. Mandell, Andrew Ujita, Jordana Henderson, Anthony Truong, Coleen Vo, Farinaz Rezvani, Nona Abolhassani, Alexandre Lebedev, Chunping Xu, Inna Koukhareva, Stephanie Ramos, Kate Broderick, Benjamin Hudson, Jeff Coller

**Affiliations:** 1 RNA Innovation Center, Institute for NanoBioTechnology, Johns Hopkins University, Baltimore, MD 21218, USA; 2 TriLink BioTechnologies, Part of Maravai LifeSciences, San Diego, CA 92121, USA

**Keywords:** MT: Delivery Strategies, mRNA, mRNA medicines, mRNA therapeutics, mRNA durability, mRNA stability, mRNA decapping, mRNA expression

## Abstract

Co-transcriptional capping allows exogenous mRNAs to yield robust protein expression. Identifying additional mRNA modifications that further boost protein output will be crucial for developing more efficacious mRNA therapies. Using *in vitro* approaches, we found that the co-transcriptional cap analog ^m7^G_3'OMe_ppp^m6^A_2′OMe_pG, CleanCap M6, resists enzymatic decapping and that this decreased susceptibility to decapping correlated with substantially increased protein expression *in vivo* compared to mRNAs capped using existing industry standards.

## Graphical abstract

**Figure:**
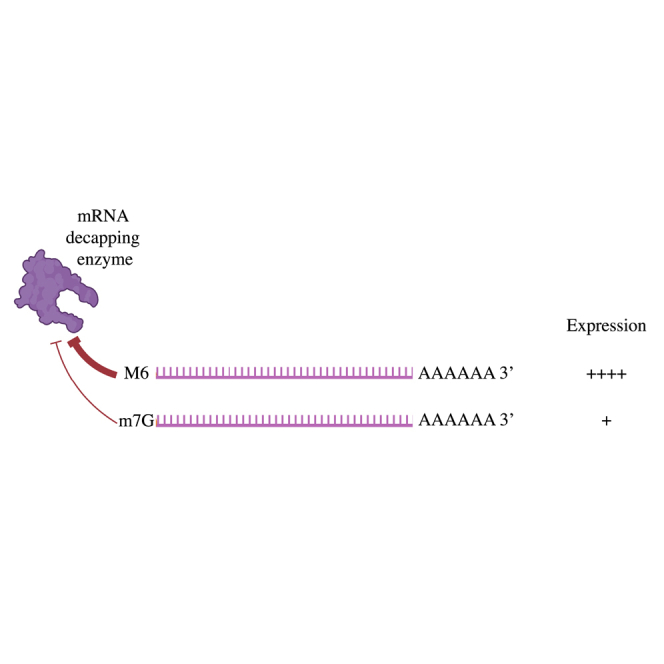

## Teaser

Mandell and colleagues present CleanCap M6, a next generation 5′ cap analog that outperforms existing industry standards in live mice. Mandell and colleagues found that this cap extends the half-life of the mRNA by inhibiting the mRNA decapping enzyme.

## Introduction

**Figure 1:**
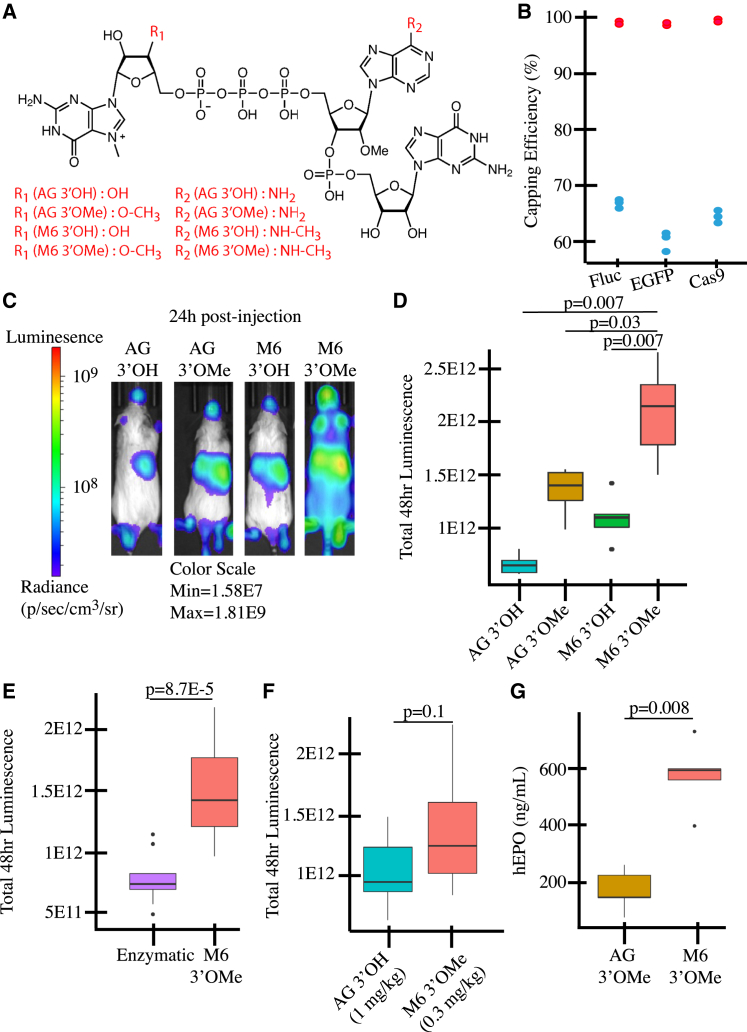
Impact of cap structure on *in vitro* and *in vivo* mRNA performance (A) Schematic of cap structures used in this study. (B) Capping efficiencies of Fluc, EGFP, and Cas9 mRNAs using Legacy (blue) or CleanCap M6 (red) IVT conditions. Each set of points consists of at least two replicates. (C) Representative whole-body luminescence image of mice injected with Fluc mRNAs capped with AG 3′OH, AG 3′OMe, M6 3′OH, or M6 3′OMe (*N* = 5). Radiance scale can be found to the left of the mice. Minimum and maximum values can be found below the mice. Bars above each pair of mice show the *p* values of Mann-Whitney comparison. (D) Total luminescence as area under curve (AUC) of mice injected with Fluc mRNAs capped with AG 3′OH, AG 3′OMe, M6 3′OH, and M6 3′OMe. (E) AUC showing mRNAs capped with M6 or capped enzymatically (*N* = 9). (F) AUC of a dose-lowering experiment (*N* = 7). (G) Protein expression from hEPO mRNAs in mouse serum at 24 h post-injection (*N* = 5).

One significant advantage of mRNA over protein-based therapeutics is that mRNA is generated enzymatically through cell-free *in vitro* transcription (IVT). Besides reducing the physical space and processing time required to generate purified drug substance, IVT allows the components of mRNA synthesis to be directly controlled, for example, by substituting modified uridine analogs or including co-transcriptional capping analogs. The 5′ cap plays a crucial role in modulating mRNA translation and decay, helping to determine the fate of the transcribed mRNA once introduced into cells.^1^ Endogenous mammalian 5′ cap structures consist of an N7-methyl-G residue attached to the first transcribed nucleotide via a 5′ to 5′ triphosphate linkage (^m7^GpppN). In addition, the first and second transcribed nucleotides can be methylated at the 2′-*O*-ribose to yield Cap1 or Cap2 structures, respectively.^1^ IVT mRNAs can be capped either co-transcriptionally or after IVT using downstream enzymes. One appeal of the co-transcriptional approach is the ability to cap the mRNA with various alternatives. For example, 3′-*O*-methylation of the m7G ribose ensures that the cap is incorporated in the correct orientation (^m7^G_3′OMe_pppN).^2^ In addition, N6-methylated adenosine at the first transcribed nucleotide of endogenous mRNA has recently garnered attention for its ability to increase protein expression through an inhibition of decapping.^3^^,^^4^^,^^5^ However, the effect of cap proximal m6A and 3′ - O - Methyl (3′OMe) on IVT mRNAs when utilized in conjunction remains unclear. In this study, we used a combination of *in vivo*, tissue culture, and *in vitro* biochemical approaches to assess the effect of mRNAs co-transcriptionally capped with ^m7^GpppA_2′OMe_pG (AG 3′OH), ^m7^G_3′OMe_pppA_2′OMe_pG (AG 3′OMe), ^m7^Gppp^m6^A_2′OMe_pG (M6 3′OH), and ^m7^G_3′OMe_ppp^m6^A_2′OMe_pG (M6 3′OMe). The structures of these cap analogs can be found in Figure 1A.

## Results

Initial experiments testing the incorporation of M6 3′OMe (now commercially known as CleanCap M6) into mRNAs using industry standard IVT protocols^6^ (see materials and methods) resulted in poor capping efficiency (60%–65%) (Figure 1B). Using a design of experiments approach, numerous conditions including pH, nucleoside triphosphate, cap, and Mg^2+^ concentrations were screened. This allowed the identification of an IVT protocol that yields high quantities of >95% capped mRNA and produces minimal double-stranded RNA (dsRNA) by-products (Figures 1B and S1A; materials and methods). Using this updated CleanCap M6 IVT condition, uridine-depleted, N1-methylpseudouridine (m1ψ)^7^-modified firefly luciferase (Fluc) mRNAs capped with M6 3′OMe or M6 3′OH were generated. Additional mRNAs that were co-transcriptionally capped with AG 3′OH (commercially known as CleanCap AG), with AG 3′OMe (commercially known as CleanCap AG 3′OMe), or capped using the vaccinia enzyme followed by 2′*O*-methyltransferase, which results in a Cap1 structure similar to AG but with a +1 and +2 G, were also generated. All mRNAs had capping efficiencies above 95% and similar dsRNA levels (Table S1).

To test the effect of these 5′ cap structures on protein expression *in vivo*, m1ψ-modified mRNAs were encapsulated into lipid nanoparticles and tail vein-injected into female CD-1 mice. Luciferase activity was then measured using whole-body bioluminescence imaging at various time points over 48 h. We found that mRNAs capped using M6 3′OMe yielded 3.1-fold more total luminescence over 48 h than mRNAs capped using AG 3′OH, 1.5-fold over AG 3′OMe, 1.9-fold over M6 3′OH, and 2.0-fold over enzymatically capped RNAs (Figures 1C–1E). In addition, serum was collected from each animal for cytokine and chemokine analysis at 3 h and 24 h post-injection, and no significant differences in the overall inflammatory profiles of the co-transcriptionally capped mRNAs were observed (Figure S1B). Purifying mRNAs by reverse phase-high-performance liquid chromatography provided no observable change in protein expression or immune responses (data not shown), confirming that the difference in potency was due to the cap structure itself. Of note, mRNAs capped with M6 3′OMe, delivered at a dose of 0.3 mg/kg, resulted in approximately the same level of expression as mRNAs capped with AG 3′OH delivered at a dose of 1 mg/kg (Figure 1F). To confirm that this effect was not luciferase specific, mRNAs encoding human erythropoietin (hEPO), a commonly used model for gene replacement therapies,^8^ were tested in C57BL6/NCrl mice. mRNAs capped with M6 3′OMe yielded roughly 3-fold more hEPO protein at 24 h, corroborating the findings from the luciferase studies (Figure 1G). Together, these findings demonstrate that the M6 3′OMe cap analog increases mRNA potency.

**Figure 2:**
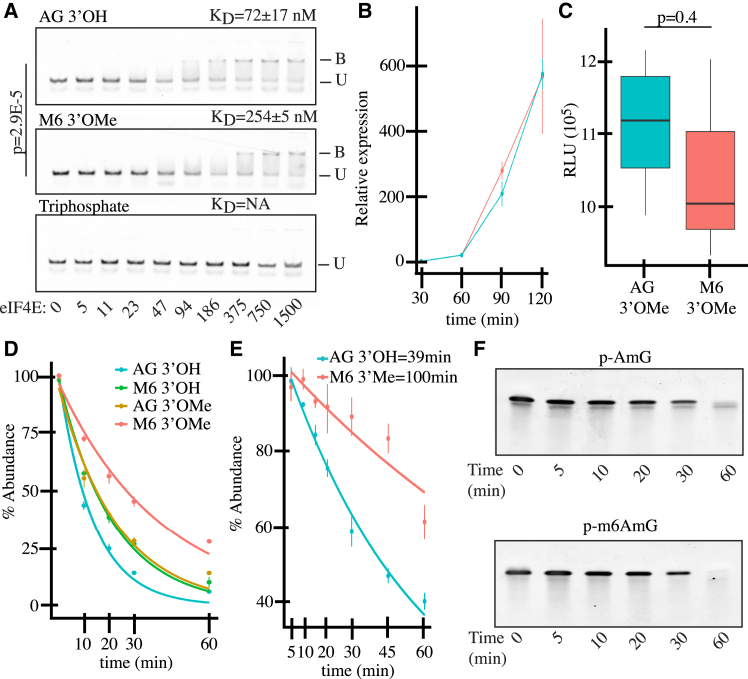
Effect of cap structure on mRNA translation and decapping (A) Quantitative gel shift assays of eIF4E bound to RNA oligos capped with AG 3′OH, M6 3′OMe, or 5′ triphosphate. The 5′ cap moiety is shown at the top left of each gel slice. The locations of the bound (B) and unbound (U) fractions are denoted by the bars to the right of the gel slices. The quantity of eIF4E can be found below the lanes of the lowest gel slice. The K_D_ values are at the top right of each gel slice. The vertical bar shows the *p* value of a Mann-Whitney comparison of the calculated K_D_ values of eIF4E for AG 3′OH vs. the K_D_ values of eIF4E for M6 3′OMe. (B) Early expression of an Fluc mRNA capped with either AG 3′OMe (blue) or M6 3′OMe (red) in HEK293T cells. For each time point, the relative expression is a measure of the relative light unit (RLU) at that time point, divided by the median RLU at t = 30 min. Error bars, mean ± standard deviation of three replicates. (C) Expression of an Fluc mRNA capped with either AG 3′OMe (blue) or M6 3′OMe (red) in rabbit reticulocyte lysates (*N* = 3). Box, first to last quartiles; whiskers, 1.5× interquartile range; center line, median. *p* value is a Student’s t test comparison. (D) Dcp2-mediated *in vitro* decapping of 43-mer oligos capped with AG 3′OH, AG 3′OMe, M6 3′OH, or M6 3′OMe. Error bars, mean ± standard deviation of three replicates. (E) Dcp2 + Xrn1 *in vitro* decay of 43-mer oligos. Error bars, mean ± standard deviation of three replicates. (F) Xrn-1 decay of monophosphate oligos by PAGE containing A_2′OMe_pG or ^m6^A_2′Ome_pG 5′ sequence.

Protein expression of exogenous mRNA is a function of both mRNA half-life and translation efficiency. Considering that translation initiation begins with eukaryotic translation initiation factor 4E (eIF4E) binding to the 5′ cap,^9^ we asked whether M6 3′OMe had a direct effect on translation initiation. To test this, purified human eIF4E^9^ (Figure S2) was incubated with increasing concentrations of RNA oligos capped with AG 3′OH or M6 3′OMe (Table S1). Using quantitative gel shifts, the equilibrium dissociation constant (K_D_) of eIF4E for both capped oligos was measured. In line with previously published results,^10^ we observed that M6 3′OMe reduces the affinity of eIF4E for the RNA relative to AG 3′OH (Figure 2A). To determine whether this impaired binding has a negative impact on translation initiation, Fluc mRNAs capped with either M6 3′OMe or AG 3′OMe were transfected into HEK293T cells. Initial luminescence changes were then measured, with the goal of measuring early translation and minimizing the impact of RNA decay. Despite a lower affinity for eIF4E (Figure 2A), M6 3′OMe did not detectably impair early translation initiation (Figure 2B). AG 3′OMe or M6 3′OMe capped Fluc mRNAs were also translated in rabbit reticulocyte lysates, which are deficient in catalytic mRNA decapping, and we found no statistically significant difference in expression (Figure 2C).

Given the lower affinity for eIF4E, we considered whether impaired decapping and thus a potentially longer mRNA half-life might be responsible for the higher protein expression seen with M6 3′OMe capped mRNAs. To explore this possibility, capped RNA oligos (Table S1) were incubated with recombinant hDcp2 protein, and the fraction of decapped RNA over time was quantified by liquid chromatography-mass spectrometry (Figure 2D). RNAs capped with AG 3′OMe and M6 3′OH were both decapped less efficiently than AG 3′OH. Moreover, combining the 3′OMe and m6A modifications showed an additive effect with even greater resistance to Dcp2-mediated decapping. Building on this system, we reconstituted a minimal *in vitro* decay system, composed of recombinant human Dcp2 and commercially available yeast Xrn1, which decays the mRNA after cap removal was tested.^11^^,^^12^ RNA oligos capped with either AG 3′OH or M6 3′OMe were treated with Dcp2 and Xrn1 across a time course, and RNA decay was quantitated by measuring the spectral shift of each time point after quenching. In this decay system, M6 3′OMe increased the half-life of the RNA by approximately 2.5-fold (Figures 2E and S3). As observed for treatment with Dcp2 alone, M6 3′OMe increased RNA half-life over M6 3′OH, AG 3′OMe, and AG 3′OH capped RNA oligos (Figure S4). To confirm the *in vitro* decay findings, we also asked whether the m6Am at the +1 position itself impacted 5′-to-3′ mRNA decay by Xrn1 following decapping, but observed no significant difference (Figure 2F).

To begin assessing whether M6 3′OMe impacts mRNA half-life directly, full-length unmodified Fluc mRNAs capped with either M6 3′OMe or AG 3′OMe, but having otherwise similar quality attributes (Figure S1A; Table S1), were transfected into HEK293T cells and mRNA decay over time quantified by quantitative reverse-transcription PCR (qRT-PCR). M6 3′OMe cap significantly increased the longevity of Fluc mRNA in HEK293T cells (up to 23-fold relative to AG 3′OMe) (Figure S6), suggesting that increased resistance to decapping by M6 3′OMe can improve mRNA longevity. While beyond the scope of the present work, future studies will explore the interplay between modified bases, including m1ψ, and modified caps, including M6 3′OMe, to determine whether additional synergistic combinations can provide even more potent effects on mRNA longevity.

## Discussion

In this study, we report that mRNAs capped with CleanCap M6 (m7G3′OMe^pppm6A^2′OMe^pG)^ yield significantly more protein *in vivo* than those capped with AG 3′OH, AG 3′OMe, or M6 3′OH and that this improvement is likely due at least in part to impaired Dcp2-mediated decapping. Thus, utilizing CleanCap M6 may help increase the potency of mRNA medicines, allowing for reduced dosing and increased efficacy in the clinic. Moreover, this research emphasizes the crucial role of mRNA stability, showing that it can be effectively modulated to enhance the effectiveness of mRNA therapeutics.

## Materials and methods

The materials and methods are found in the supplemental information.

## Data and code availability

The data underlying this article are available in the article and in its supplemental information.

## Acknowledgments

The authors thank members of the Coller lab for helpful discussion. The work was supported by a grant from the 10.13039/100000002National Institutes of Health (R35GM144114 to J.C.) and TriLink BioTechnologies, part of Maravai LifeSciences.

## Author contributions

Z.F.M., A.U., J.H., B.H., and J.C. wrote the manuscript. Z.F.M., A.U., A.T., C.V., F.R., N.A., A.L., C.X., I.K., and B.H. conducted all experiments. J.C., J.H., B.H., S.R., and K.B. oversaw all experimental designs and analyses.

## Declaration of interests

J.C. was funded in part by a grant from Maravai LifeSciences, parent company of TriLink BioTechnologies. Authors noted as being affiliated with TriLink BioTechnologies are employees of TriLink BioTechnologies, a subsidiary of Maravai LifeSciences Holdings, and are shareholders thereof. These authors may also be inventors on patents related to this work, a list of which is available at https://www.trilinkbiotech.com/legal-notices.

## Supplemental information

Document S1. Figures S1–S6, Table S1, and supplemental materials and methods

Document S2. Article plus supplemental information
