# Supplementary material for: CleanCap M6 inhibits decapping of exogenously delivered IVT mRNA
Source: Mol Ther Nucleic Acids. 2025 Jan 17;36(1):102456. doi: 10.1016/j.omtn.2025.102456 (PMC11835620; doi:10.1016/j.omtn.2025.102456)
Supplement: Document S1. Figures S1–S6, Table S1, and — supplemental materials and methods [file mmc1.pdf]

OMTN, Volume *36*

Supplemental information

CleanCap M6 inhibits decapping

# of exogenously delivered IVT mRNA

Zachary F. Mandell, Andrew Ujita, Jordana Henderson, Anthony Truong, Coleen Vo, Farinaz Rezvani, Nona Abolhassani, Alexandre Lebedev, Chunping Xu, Inna Koukhareva, Stephanie Ramos, Kate Broderick, Benjamin Hudson, and Jeff Coller

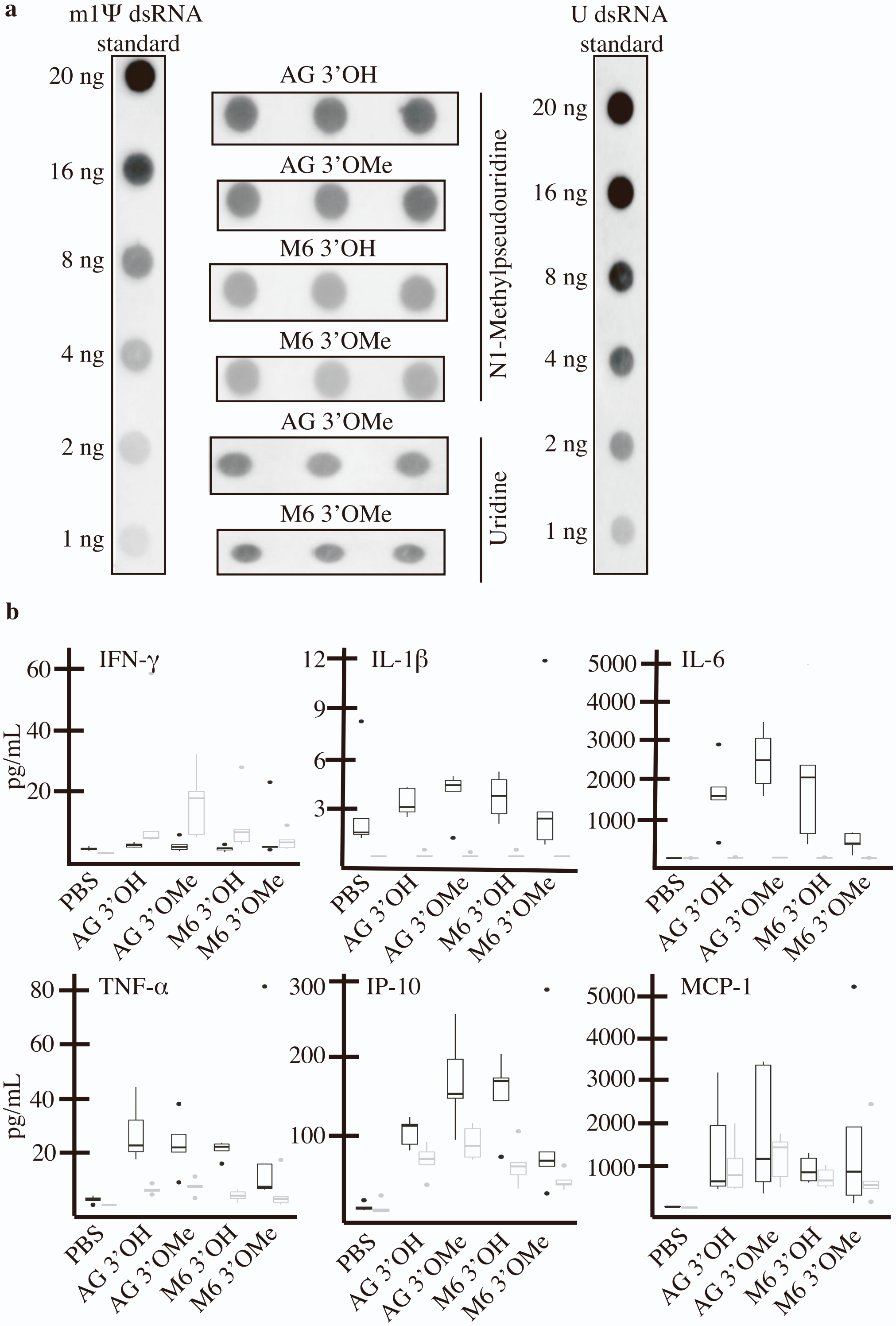

Figure S1. Comparison of dsRNA and immunology data from cap structures. a. Dot blots showing the amount of dsRNA formation during the IVT reaction when using AG 3'OH, AG 3'OMe, M6 3'OH, and M6 3'OMe caps. dsRNA standards can be found in the slice to the left for N1-methylpseudouridine, and unmodified (uridine) bases on the right while reaction products can be found in the middle loaded as 2 μg per dot in technical triplicate. **b.** Serum cytokines and chemokines measured 3 hours (black) and 24 hours (grey) after injection. For each graph, the measured biomolecule can be found on the top left of the graph (N=5 animals per Figure 1c-d).

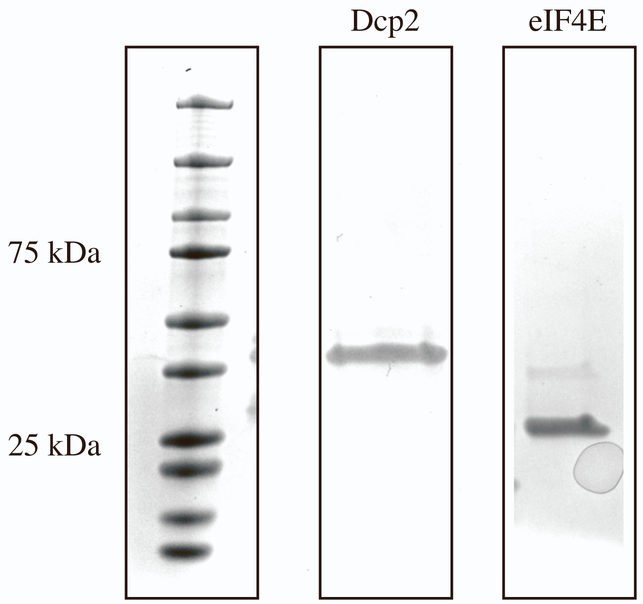

Figure S2. Purity of eIF4E and Dcp2 protein preps. Coomassie stained SDS-PAGE gels showing the Dcp2 (middle gel slice) and eIF4E (right gel slice) protein preps. The molecular ladder can be found in the gel slice to the left.

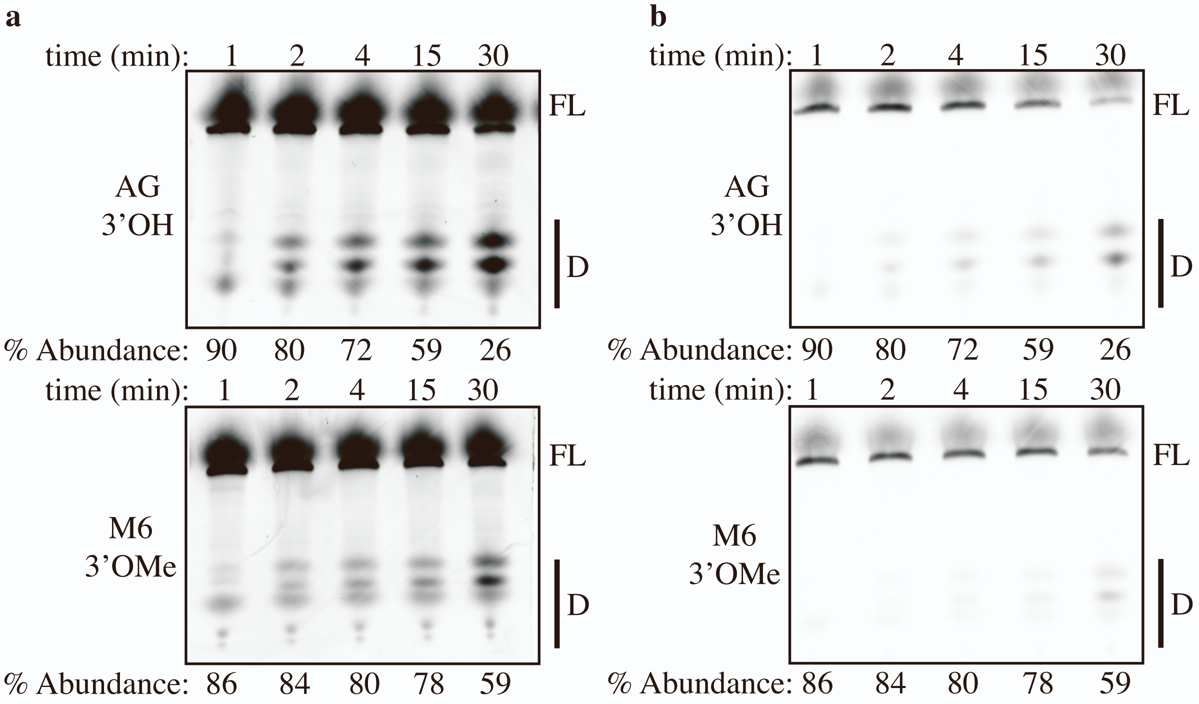

Figure S3. M6 inhibits Dcp2 mediated decapping *in vitro*. PAGE-resolved products of the in vitro decay assay. The 5' cap moiety can be found to the left of each gel slice. The locations of the decayed (D) and full-length (FL) RNA species are denoted to the right of the gel slices. The % abundance of the full-length product can be found below each lane. The time-point can be found above each lane. **a.** Long exposure to detect decay and **b.** intensity set to ensure that the signal of the full-length product was in the linear range of same gel image (N=1).

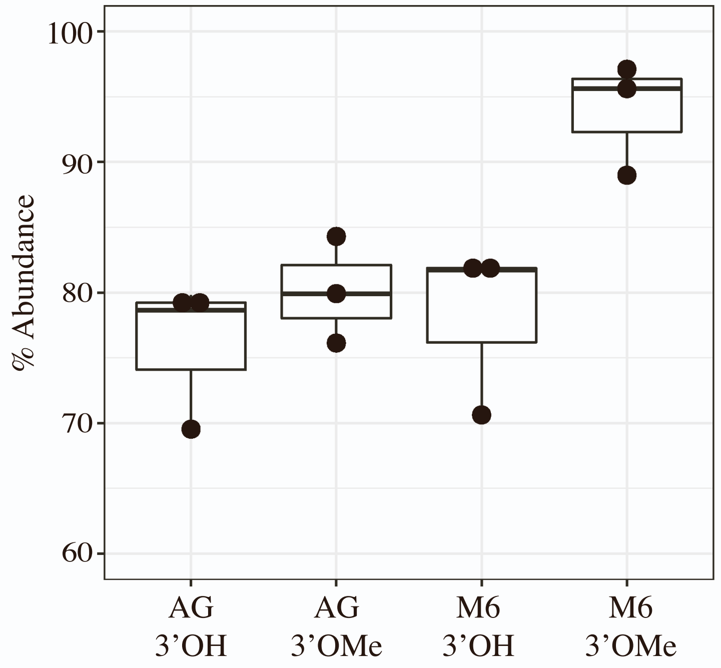

Figure S4. M6 3'OMe outperforms AG 3'OH, AG 3'OMe, and M6 3'OH *in vitro*. The % abundance of RNAs capped with AG, AG 3'OMe, M6 3'OH, or M6 3'OMe after a 15-minute incubation with the minimal decay system composed of Dcp2 and Xrn1.

a

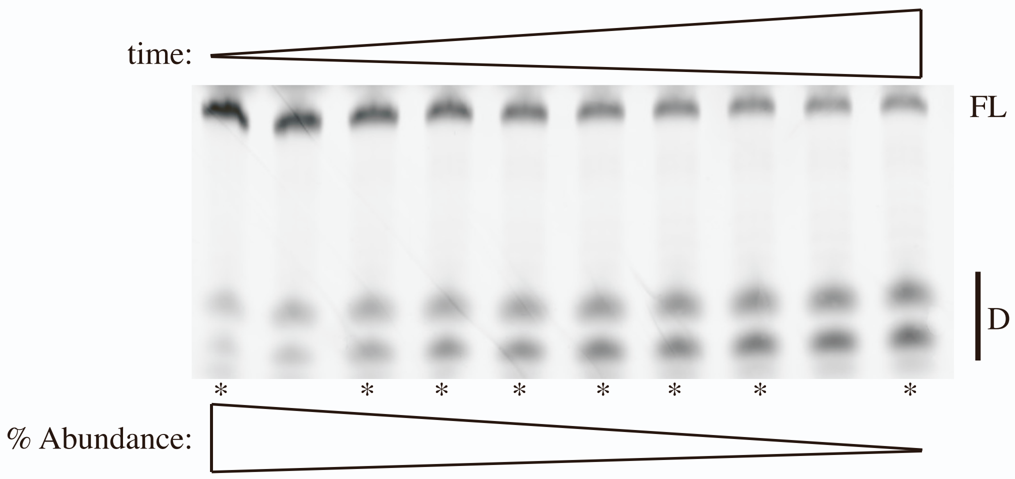

b

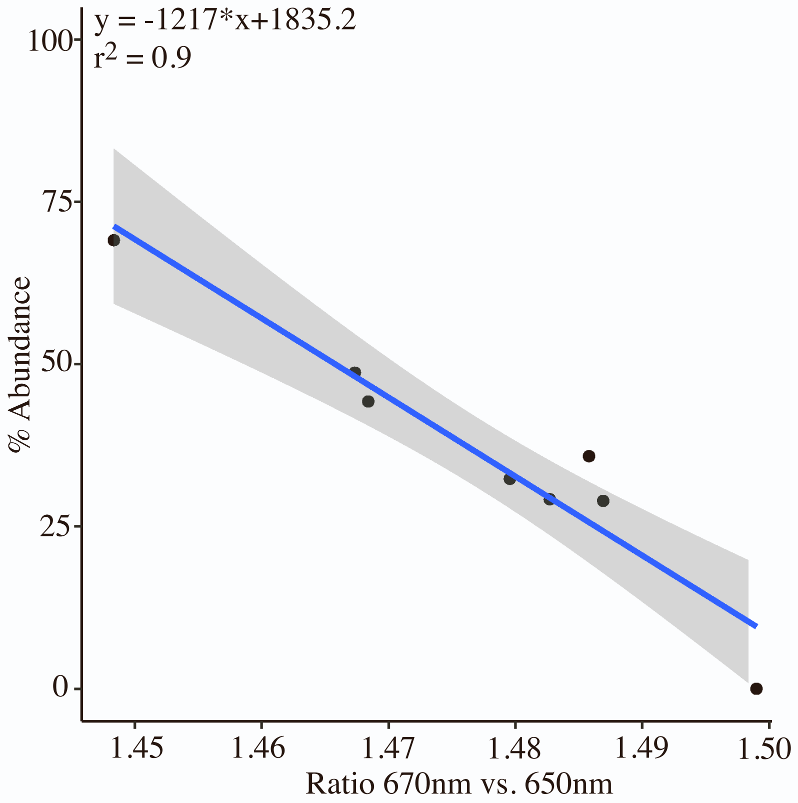

Figure S5. Standard curve for *in vitro* decay system. a. Products of the RppH and Xrn1 containing minimal decay system across the time course. The locations of the decayed (D) and full-length (FL) uncapped RNA oligo species are denoted to the right of the gel slices. The wedge below the lane shows the relative amount of the full-length product across the line course. **b**. The % abundance of the full-length RNA species vs. the spectral shift of that RNA pool. The blue line shows the linear regression, with the grey shadow being the standard error of the linear regression. The linear regression itself and the r^2 value can be found in the top left of the plot.

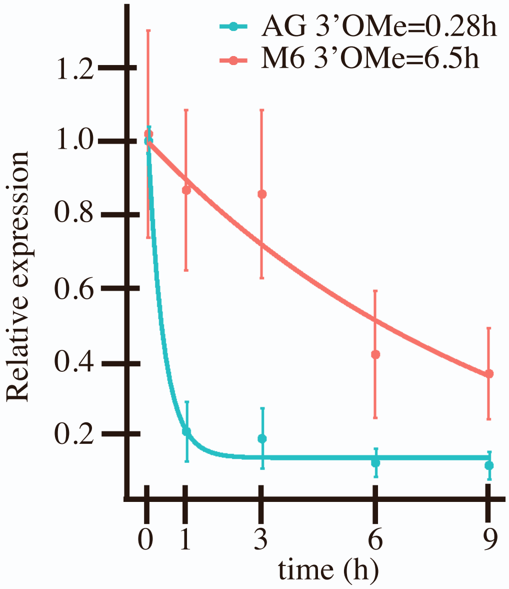

Figure S6. mRNA half-life in cells. Decay curves of unmodified (WT uridine) Fluc mRNAs capped with either AG 3'OMe (blue) or M6 3'OMe (red) 2h post-transfection in HEK293T. Error bars, mean value ± standard deviation of at least 2 replicates.

Table S1. RNA quality attributes

Sample	IVT Method (TriLink Cat. No.)	mRNA Quality (analysis technique)	Capping Efficiency (LCMS)	dsRNA (dotblot)	Experiment
N1mePsU Fluc, AG 3'OH Cap	CleanScript*(Custom synthesis of L-7602)	88% (IPRPHPLC)	97.9%	~6.5 ng/µg	CD1 mice (Genvoy LNP)
N1mePsU Fluc, AG 3'OMe Cap	CleanScript*(Custom synthesis of L-7602)	94.7% (IPRPHPLC)	96.7%	~5.8 ng/µg	CD1 mice (Genvoy LNP)
N1mePsU Fluc, M6 3'OH Cap	CleanCap M6	96.9% (IPRPHPLC)	98.4%	~3.2 ng/µg	CD1 mice (Genvoy LNP)
N1mePsU Fluc, M6 3'OMe Cap	CleanCap M6 (L-8102)	96.7% (IPRPHPLC)	97.1%	~3.6 ng/µg	CD1 mice (Genvoy LNP)
N1mePsU Fluc, Enzymatic Cap	Uncapped + Vaccinia and 2'OMe transferase	90.8% (IPRPHPLC)	99.8%	~9 ng/µg	CD1 mice (Genvoy LNP)
N1mePsU hEPO, AG 3'OMe Cap	CleanScript* (Custom synthesis of L-7209)	86.7% (IPRPHPLC)	96.5%	<1 ng/µg	C57BL6/NCrL mice (Genvoy LNP)
N1mePsU hEPO, M6 3'OMe Cap	CleanCap M6 (L-8109)	96.6% (IPRPHPLC)	97.7%	<1 ng/µg	C57BL6/NCrL mice (Genvoy LNP)
WT Fluc, AG 3'OMe Cap	CleanScript*(Custom synthesis of L-7602)	88.4% (Fragment Analyzer)	n.m. (>95% per average result)	~3.5 ng/µg	HEK293T, RRL
WT Fluc, M6 3'OMe Cap	CleanCap M6 (Custom synthesis of L-8102)	91.8% (Fragment Analyzer)	n.m. (>95% per average result)	~3.4 ng/µg	HEK293T, RRL
AG 3'OH cap oligo	Custom	99.25% (LCMS)	100%**	N/A	eIF4e, DCP2, and/or Xrn-1
AG 3'OMe cap oligo	Custom	99.46% (LCMS)	100%**	N/A	eIF4e, DCP2, and/or Xrn-1
M6 3'OH cap oligo	Custom	98.16% (LCMS)	100%**	N/A	eIF4e, DCP2, and/or Xrn-1
M6 3'OMe cap oligo	Custom	99.44% (LCMS)	100%**	N/A	eIF4e, DCP2, and/or Xrn-1

Abbreviations: n.m. = not measured; N/A = not applicable
*Proprietary optimized IVT conditions to TriLink available through CDMO services
**No uncapped species detected
dsRNA values are approximate by semi-quantitative nature of method relative to ds335 standard with matched uridine base (N1mePsU or WT; wildtyp

Methods

RNA Synthesis and analysis

All RNA was made or supplied by TriLink using methods developed at TriLink.

Uridine depleted Firefly luciferase or human EPO mRNA was transcribed from a PCR-generated DNA template encoding a T7 promoter, "AG" at the +1 and +2 positions, mammalian 5' and 3' UTRs, and a 120A/T track. mRNAs were transcribed with N1-methyl-Pseudo UTP for animal studies or wild-type UTP for in vitro and cell studies. All cap and NTP reagents were provided by TriLink Biotechnologies (San Diego, CA). CleanCap M6 RNAs were either purchased as off-the-shelf catalog items, such as L-8102 (FLuc), or ordered through TriLink custom RNA synthesis. All CleanCap M6 RNAs were transcribed according to the following IVT conditions: 5 mM each NTP (A,C,G, U or N1mePsU), 10mM CleanCap M6, 40 mM Tris-HCl pH 7.5, 15mM (additional) HCl, 15 mM magnesium chloride, 10 mM DTT, 2mM spermidine, 0.025 µg/µL DNA template, 1 U/µL Rnease Inhibitor, 0.002 U/µL inorganic pyrophosphatase, and 15 U/uL T7 RNA polymerase at 37°C for 3 hours.

Control CleanCap AG and AG 3'OMe mRNAs were synthesized by TriLink using either "legacy" industry-standard conditions (5mM each NTP, 4mM CleanCap, 40 mM Tris pH 8, 16.5 mM magnesium acetate, 10mM DTT, 2mM spermidine, 0.002% Triton-X, 0.025 µg/µL DNA template, 1 U/µL Rnase Inhibitor, 0.002 U/µL inorganic pyrophosphatase, and 8 U/µL T7 RNA polymerase at 37°C for 3 hours) for capping efficiency comparisons or a dsRNA-optimized transcription condition (CleanScript IVT) which is proprietary to TriLink Biotechnologies but available via off-the-shelf products (L-7602, Fluc, WT, CleanCap AG) or custom request for other caps, sequences, and base modifications.

Following IVT incubations, DNaseI and respective buffer were used to hydrolyze starting DNA template. Reaction samples were then treated with 0.1 mg/mL of proteinase K purchased from Sigma-Aldrich (St. Louis, MO) for 1 hour at 37 ºC in the presence of 0.5% SDS and 5 mM DTT to digest all previously added enzymes down to polypeptides prior to clearance by tangential flow filtration (TFF) through 10 diavolumes on an Omega PES 100 kDa MWCO membrane by Pall (Port Washington, NY) . All mRNAs were affinity purified by oligo dT chromatography to clear untailed fragments and remaining reaction components. Oligo dT chromatography was performed on an AKTA Avant 25 (Cytiva) with an 8 mL BIA CIMultus column (Ajdovscina, Slovenia) with flow rate of 3 column volumes per minute (CV/min). Samples were then buffer exchanged by TFF for final 1 mg/mL concentration in 1 mM sodium citrate, pH 6.4 and sterile filtered by 0.2 µm membrane.

Crude mRNA yields were assessed by analytical IP-RP-HPLC against a standard curve or LiCl precipitation and UV-Vis spectrophotometry. All purified mRNA yields were measured by UV-Vis spectrophotometry with peak maximums at 260 nm. mRNA integrity was analyzed by IP-RP-HPLC using a Thermo DNAPac RP 3.0 x 50 mm column (Part # 088920). Mobile phase A was 100 mM TEAA/ 1 mM EDTA pH 6.5-7, and mobile phase B was MPA with 25% acetonitrile. 1 µg of mRNA sample was analyzed at 65 C with a gradient from 40 to 60% B over 14 minutes at 0.3 mL/min. Capping efficiency was assessed by LC-MS analysis of cleaved 5' mRNA fragments using TriLink's in-house analytical method. The estimation of capping efficiency was made using the following formula. Capping efficiency % = Σ Mass Intensities of identified 5' capped species/(Σ mass intensities of identified 5' capped + 5' uncapped species)x100. Relative dsRNA was measured by immunoblot using TriLink made dsRNA standards and J2 antibody[13] with 2 µg of test sample RNA loaded onto membrane per dot, technical triplicates assessed.

LNP formulation

All mRNA samples were encapsulated into GenVoy-ILM lipid nanoparticles using NxGen microfluidic platform (Precision NanoSystems, Vancouver BC) targeting 0.5 μg/μL mRNA concentration.

In vivo delivery

The Charles River Laboratories' Institutional Animal Care and Use Committee (IACUC) reviewed and approved all Fluc mRNA:LNP procedures involving mice. Female CD-1 mice of 8-10 weeks old were randomized into groups of 5 animals based on body weight at the beginning of the study (Charles River Laboratories, Discovery Research Services North Carolina). LNP:mRNA test articles were diluted in PBS to achieve 1 mg/kg delivery in a single bolus by tail-vein injection. The body weight of each mouse was measured once a day for the duration of the study and 2 blood draws were taken for serum analysis by Mouse Cytokine/Chemokine 26-Plex ProcartaPlex Panel 1 by Luminex platform (Thermo Fisher). Luciferase activity was measured by whole-body bioluminescence imaging on IVIS Spectrum CT system by Perkin Elmer (Greenville, SC) at 7 time points post mRNA injection (3, 6, 9, 12, 24, 36, and 48 hours) with D-luciferin intraperitoneal injection 10 minutes prior to imaging sessions (150 mg/kg total).

hEPO mRNA studies were conducted at HDBiosciences, San Diego, CA. Female C57BL6/NCrl mice aged 9-10 weeks were randomized into treatment groups of 5 animals based on body weight on Day 1 of study. LNP:mRNA test articles were delivered by IV injection at 0.1 mg/kg.

Serum was collected for ELISA analysis using Human Quantikine IVD Elisa Kit by R&D Systems (Catalog# DEP00).

Statistical analyses of animal studies

Each pair student's t-tests were performed in JMP (Version 15.1, Cary, NC) to compare differences between mouse cohorts.

Preparation of capped RNA oligos

Capped 43-mer oligo RNAs were transcribed from short DNA templates with only a single templated cytidine at the very 3' position (sequence below). RNAs were transcribed with a single 3'-terminal Cy5-CTP for Dcp2/Xrn1 coupled spectral shift assays or with wild-type CTP for LC-MS decapping assays. The RNA oligos were prepared in 200 μL in-vitro transcription reactions. After incubation, IVT reactions were quenched by addition of 0.5 M EDTA to 50 mM and purified by phenol-chloroform and 1-bromo-3-chloropropane extractions to remove protein. After organic extraction, each IVT reaction was purified by RP-HPLC to isolate capped full-length transcripts from uncapped and abortive transcripts. HPLC purification utilized an AKTA Avant 25 FPLC system and a 10 x 150 mm Hamilton PRP-H1 C18 column (Hamilton part # 79266) heated to 65 C. The mobile phases were 100 mM TEAA and 100 mM TEAA + 40% acetonitrile, both at pH 7. Full-length transcripts elute in a single broad peak in a gradient from 20 to 35% B over 7.5 CV. Capped and uncapped transcripts separate within this peak, which was collected in several fractions. Fractions were analyzed by LC-MS and those containing >95% capped FLP were pooled, then exchanged into 1 mM sodium citrate pH 6.4 and concentrated over 3 kDa Amicon spin filters.

43mer oligos from preparative HPLC fractions and decapping reactions were analyzed by LC-MS using a Thermo Scientific Orbitrap Exploris 120 mass spectrometer coupled to a Thermo Scientific Vanquish Horizon UHPLC and a 2.1 x 50 mm Acquity UPLC BEH C18 column (part # 186002350) heated to 65 C. Mobile phase A was 0.1% hexafluoroisopropanol/ 0.1% diisopropylethylamine/ 2 µM EDTA, mobile phase B was 100% LC-MS grade acetonitrile, and the LC method delivered a gradient from 2 to 25% B over 7.5 min at 300 µL/min. Intact mass analysis was performed in the negative heated electrospray ionization mode at a resolution of 120,000 with a 400-2000 *m/z* scan range. All mass spectra were deconvoluted using ProMass (Novatia).

Sequence of RNA oligo

5’ AGGAAAUAAGAGAGAAAAGAAGAGUAAGAAGAAAUAUAAGAGC 3’

Protein purification

The Dcp2 and eIF4E coding sequences were codon-optimized for bacterial expression using the GenScript codon optimization tool. Briefly, a gBlock^{TM} DNA fragment was purchased, which contained an NheI cleavage site, followed by a start codon, then a 6-histidine-protein G tag, then a TEV protease cleavage site, then the optimized Dcp2 or eIF4E sequence, and ended with a XhoI cleavage site. This DNA fragment was PCR amplified using Vent Polymerase and then cloned into the pET-28c bacterial overexpression vector between the NheI and XhoI sites. Upon confirmation of correct insertions, the Dcp2 and eIF4E overexpression vectors were transformed into BL21 (DE3) cells.

Both Dcp2 and eIF4E were purified as described previously[14,15]. To purify Dcp2 or eIF4E, the BL21 strain expressing the pET28c vector containing the appropriate sequence was streaked out onto LB plates containing 30 µg /mL Kanamycin. The next day, a single colony was picked and used to inoculate 5 mL of LB liquid media supplemented with 30 µg /mL of Kanamycin. This culture was grown overnight shaking at 37°C. The next morning, 2.5 mL of the overnight growth was added to 250 mL of pre-warmed TB media supplemented with 30 µg/mL of Kanamycin. This flask was grown with shaking at 37°C until the O.D. reached 0.4-0.6. At this point, the flask was taken out of the incubator and left standing at room-temperature for approximately 10 minutes, at which point IPTG was added to 1 mM for Dcp2, and 0.5 mM for eIF4E. The flask was then shaken at 19°C for ~18-24 hours. After this time, the cell culture was centrifuged for 20 minutes at 4000 rpm at 4°C. The media was poured off and the wet bacterial pellet was stored at -80°C for at least 20 minutes. The frozen cell pellet was then resuspended in either Dcp2 Lysis Buffer (500 mM NaCl, 40 mM Tris pH 7.9, 2 mM β-ME, 5% Glycerol, 25 mM Imidazole, 1% Triton X-100, 0.5 M Urea, 0.5 mg/mL Lysozyme, 1 pill of Roche EDTA Free protease inhibitor cocktail per 50 mL Lysis Buffer) or eIF4E Lysis Buffer (200 mM KCl, 20 mM HEPES pH 7.5, 1 mM DTT, 10% Glycerol, 25 mM Imidazole, 0.5 mg/mL Lysozyme, 1 pill of Roche EDTA Free protease inhibitor cocktail per 50 mL Lysis Buffer). The slurry was kept on ice for 30 minutes. During this time, the French-press was prepped. After the 30 minutes had passed, the cell slurry was passed through the ice-cold French-press 3 times at ~1500psi. The crude lysate was then spun down for 20 minutes at 30,000g. The clarified lysate was quickly removed from the pellet and added to Ni-NTA resin that had been pre-equilibrated with the appropriate Lysis Buffer. The bead slurry was mixed gently at 4°C for 1 hour, then added to an empty poly-prep chromatography gravity-flow column. Once all of the beads had settled, the beads were washed with 10 column volumes of the Dcp2 Lysis

Buffer, followed by 10 column volumes of either Dcp2 wash buffer (200 mM NaCl, 40 mM Tris pH 7.9, 2 mM β-ME, 5% Glycerol, 25 mM Imidazole), or 20 volumes of eIF4E Lysis Buffer. The proteins were then eluted using either Dcp2 elution buffer (Wash Buffer with 250 mM Imidazole), or eIF4E elution buffer (Lysis Buffer with 250 mM Imidazole). One mL elution fractions were collected and Bradford reagent was used to gauge where protein elution started and stopped. The eluent was collected and then, dialyzed overnight at 4°C into the appropriate elution buffer supplemented with ~60 units TEV protease. In the morning, the eluate was added to Ni-NTA resin that had been pre-equilibrated with either Dcp2 Wash Buffer or eIF4E Lysis Buffer and the slurry was mixed gently at 4°C for 1 hour, then added to an empty poly-prep chromatography gravity-flow column. The flow-through was collected and then concentrated to 0.5 mL using a Pierce PES protein concentrator and then the buffer was switched to a low-salt cation exchange buffer (50 mM KCl, 20 mM HEPES pH 7.3, 10% Glycerol, 1 mM DTT, and 0.05% Tween-20 for Dcp2) using a PD10 column. The protein was then captured via FPLC using a HiTrap SP HP cation exchange column and eluted using a gradient of cation exchange elution buffer (1 M KCl, 20 mM HEPES pH 7.3, 10% Glycerol, 1 mM DTT, and 0.05% Tween-20 for Dcp2). The fractions corresponding to the largest elution peak were collected and concentrated to 0.5 mL using a Pierce PES protein concentrator and the buffer was switched to storage buffer (200 mM KCl, 20 mM HEPES pH 7.3, 10% Glycerol, 1 mM DTT, and 0.05% Tween-20 for Dcp2). The concentration of the purified protein was measured using a DC assay and the purity was gauged via SDS-PAGE with the protein bands being visualized using a Coomassie stain. The protein preparation was then aliquoted for single-use, flash frozen using LN2 and stored at -80°C.

Gel shift assays and analysis

The gel-shift assays were conducted as described previously[16], with several modifications. 25 nM of oligo was incubated with a 2-fold serial dilution of eIF4E starting at 1.5 μM and ending at 5 nM. All of these binding events were held in a binding buffer (100 mM NaCl, 50 mM Tris-Cl pH 7.9, 10mM $MgCl_2$, 1 mM DTT, 100 μg/ml BSA, 2ng/μL Heparin, and 5% Glycerol). The various species were resolved on a 6% non-denaturing PAGE gel buffered with TBE. The gels were first run for 5 minutes at 75V at 24°C, after which they were run for 20 minutes at 150V at 4°C. The products were visualized using an Amersham Typhoon Phophor imager and digitally captured using the associated Amersham Typhoon software. The relative intensity of the unbound fraction was quantified using ImageJ and the K_D were calculated using the following model, which assumes 1:1 binding. $Y = Bmax*x/(K_D+x)$, where Bmax is the maximum binding value, while x is the concentration of eIF4E.

In-vitro decapping assays by LC-MS

A working hDCP2 stock containing 0.4 μM hDCP2 and 100 μg/mL rAlbumin (NEB) in the enzyme storage buffer described above was prepared immediately before in-vitro decapping. 20 μL decapping reactions containing 2 μM 43mer RNA, 80 nM hDCP2, and 1X mRNA Decapping Enzyme reaction buffer (NEB) were assembled in triplicate and incubated at 37 C for 0, 10, 20, 30, or 60 min, then quenched by addition of 0.5 M EDTA to 65 mM. Quenched reactions were QSed to 50 μL with LC-MS MPA, then injected in their entirety for the LC-MS analysis described above.

%Decapping was determined by summing the intensities of all deconvoluted masses corresponding to decapped FLP and variants thereof, e.g. sodium adducts. This sum is divided by

the sum of all intensities corresponding to capped and decapped FLP species and their respective variants.

Xrn1 decay of decapped oligos

5'-p-Am and 5'-p-m6Am 43-mer oligos were synthesized by TriLink Biotechnologies custom oligo synthesis service and were prepared by conventional oligo synthesis techniques. For Xrn1 decay, 2 µM oligos were incubated with 0.002 U/µL yeast XRN-1 (NEB cat # M0338L) in NEB r3.1 buffer in a 100 µL reaction. 10 µL time points were taken and immediately quenched with 2x TBE-Urea sample buffer (ThermoFisher). For analysis, 5 µL of heat denatured RNA/1x TBE-Urea was loaded onto a 15% TBE-Urea (ThermoFisher) with a target of 5 pmol oligo per band for t_0 samples. Gels were imaged and quantified on a ThermoFisher iBright CL1500 imaging system.

Spectral shift, decapping assay, and analysis

For Dcp2/Xrn1 coupled decapping assays, 20 nM of RNA oligo was incubated with 500 nM of purified Dcp2 and 0.5 units of Xrn1 (NEB cat # M0338L) in a decapping reaction buffer (100 mM NaCl, 50 mM Tris-Cl pH 7.9, 10 mM $MgCl_2$, 1 mM DTT, 100 µg/ml BSA) at 37°C for the specified time-points. To quench the reaction for PAGE, the mixture was added to 2X RNA stop solution/loading dye (40 mM Tris-base, 20 mM Na2EDTA, 0.2% sodium dodecyl sulfate, 0.05% bromophenol blue and 0.05% xylene cyanol in formamide). To quench the reaction for spectral shift, SDS was added to 5% and the mixture was boiled for 5 minutes. In cases where RppH was tested, the same general reaction conditions were used and 0.005 units of RppH (NEB cat # M0356S) were added to the reaction. To resolve the capped from the uncapped RNA species, the

mixture was loaded onto either a 15% polyacrylamide 8M Urea sequencing gel and run for 16 hours at 300V, or an 8% denaturing PAGE gel and run for 30 minutes at 200V. The products were visualized using an Amersham Typhoon Phophor imager and digitally captured using the associated Amersham Typhoon software. To quantify the % abundance via PAGE, the intensity of the full-length and the decayed RNA species were quantified using ImageJ and these intensities were compared to each other (non-decayed / decayed + non-decayed). Spectral shift was conducted using the following method. Each sample was loaded into premium glass capillary tubes and the set of tubes was fed into a NanoTemper Monolith X. The fluorescence intensity at 670nm and 650nm were measured. The ratio of these intensities was then calculated. The RNA half-life was quantitated using the following model. [% abundance] = $C*(e^{k*t})$, where C is the minimum % abundance value, k is the rate of decay, and t is time. To find the half-life, -0.693 is divided by k, where -0.693 is the natural log of 2.

To establish a standard curve for this assay, we reconstituted a minimal *in vitro* decay system, composed of RppH[17], a bacterial 5' pyrophosphohydrolase, and yeast Xrn1. In this system, RppH removes a terminal pyrophosphate from a 5’ triphosphate RNA, thereby unveiling a 5’ monophosphate that is sensitive to Xrn1. After incubating this mixture across a time-course, we split each time-point in two. For one pool, we measured the % abundance of the full-length RNA via denaturing PAGE (Fig. S5a). For the second pool, we measured the spectral shift. Indeed, we found that the spectral shift and the % abundance followed a linear relationship, with an r^2 value of 0.9 (Fig. S5b).

Cell culture, mRNA transfection, luminescence measurement, qPCR, and analyses

Human embryonic kidney (HEK) HEK293T cells were cultured in DMEM supplemented with 10% fetal bovine serum in an incubator at 37 °C with 5% CO2. Prior to transfection of unmodified Fluc mRNA (WT bases), cells were seeded onto 6-well plates, with 0.4*10^6 cells per well. Once the cells had reached 60-70% confluency, each well was transfected with mRNA at around 40k copies of each mRNA per cell. Transfections were conducted using the Mirus mRNA transfection kit using the standard conditions. For the half-life experiment, the cells were incubated with the transfection reagents for 2 hours, after which the cells were washed with 1X PBS, and fresh media was applied. At the specified time-points after initial transfection, the media was removed and total RNA was extracted using Trizol reagent. cDNA was generated using Protoscript II, 250ng of total RNA, and random hexamer primers, with a 60-minute extension time at 42°C. Each cDNA mixture was then diluted 5-fold and the diluted cDNA was then used as template in a qPCR reaction. Each qPCR was conducted using the KAPA SYBER FAST qPCR kit, with forward and reverse primers against Fluc and Actin at concentrations of 400nM each at 0.5 µL of diluted cDNA. Ct values were generated using absolute quantitation. mRNA half-life was determined by first determining the relative expression of Fluc across the time-course, with the relative expression equal to 2^-(Ct^0-Ct^t), where Ct^0 is the median Ct value at time 0, and Ct^t is the median C^t value at a particular time point. Fluc C^t values were excluded from the analysis when the median C^t value of Actin for that biological replicate was greater than 1 standard deviation away from the median C^t value of Actin across all replicates. The mRNA half-life was quantitated using the following model. [relative abundance] = C*(e^{k*t}), where C is the minimum relative abundance value, k is the rate of decay, and t is time. To find the half-life, -0.693 is divided by k, where -0.693 is the natural log of 2. For the translation efficiency experiment, the cells were incubated with the transfection reagents for the specified time-points, at which point the cells were washed with 1X PBS, scraped off the plate,

pelleted, and lysed using Promega lysis buffer in the Luciferase assay system kit. Luciferase activity was then measured using the Luciferase assay system kit, with RLU values obtained using a GLOMAX 20/20 Luminometer. For each time-point, the relative expression was obtained by dividing the RLU at that time point with the median RLU at earliest time-point.

Rabbit reticulocyte experiments

The rabbit reticulocyte experiments were conducted as follows. 20 ng of luciferase mRNA was incubated with 3.5 μL of nuclease treated lysate in a final volume of 5 μL at 30°C for 60 minutes (N= 3). After this time, the reactions were stopped by flash-freezing the mixture on dry-ice. Each reaction was then diluted with 95 μL of 1 mg/ml BSA and 2 μL of each reaction was mixed with 25 μL of the luciferase assay reagent. RLU values obtained using a GLOMAX 20/20 Luminometer.
